# Chronic alcohol exposure promotes HCC stemness and metastasis through *β*-catenin/miR-22-3p/TET2 axis

**DOI:** 10.18632/aging.203059

**Published:** 2021-05-21

**Authors:** Danlei Chen, Yan Yan, Xinyi Wang, Suzhi Li, Yan Liu, Dandan Yu, Yongjing He, Ruiqing Deng, Yakun Liu, Mei Xu, Jia Luo, Hongjun Gao, Siying Wang

**Affiliations:** 1 School of Basic Medical Sciences, Anhui Medical University, Hefei 230032, Anhui, China; 2 The First Affiliated Hospital of USTC, Division of Life Sciences and Medicine, University of Science and Technology of China, Hefei 230026, Anhui, China; 3 Department of Pulmonary Oncology, The Fifth Medical Center of Chinese PLA General Hospital, Fengtai, Beijing 100071, China; 4 Department of Pharmacology and Nutritional Sciences, University of Kentucky, College of Medicine, Lexington, KY 40536, USA; 5 Department of Pathology, University of Iowa Carver College of Medicine, Iowa City, IA 52242, USA

**Keywords:** HCC, metastasis, stemness, alcohol

## Abstract

Hepatocellular Carcinoma (HCC) patients usually have a high rate of relapse and metastasis. Alcohol, a risk factor for HCC, promotes the aggressiveness of HCC. However, the basic mechanism is still unclear. We used HCC cells and an orthotopic liver tumor model of HCC-LM3 cells for BALB/C nude mice to study the mechanism of alcohol-induced HCC progression. We showed that chronic alcohol exposure promoted HCC cells metastasis and pulmonary nodules formation. First, we identified miR-22-3p as an oncogene in HCC, which promoted HCC cells stemness, tumor growth, and metastasis. Further, we found that miR-22-3p directly targeted TET2 in HCC. TET2, a dioxygenase involved in cytosine demethylation, has pleiotropic roles in hematopoietic stem cells self-renewal. In clinic HCC specimen, TET2 expression was not only decreased by alcohol consumption, but also inversely correlated with miR-22-3p levels. Then, we demonstrated that TET2 depletion promoted HCC cells stemness, tumor growth and metastasis. Furthermore, we identified that *β*-catenin was an upstream activator of miR-22-3p. In conclusion, this study suggests that chronic alcohol exposure promotes HCC progression and *β*-catenin/miR-22-3p/TET2 regulatory axis plays an important role in alcohol-promoted HCC malignancy.

## INTRODUCTION

Hepatocellular carcinoma (HCC) has the fifth highest incidence in the United States [1] and the second highest incidence in China [2, 3]. HCC patients are usually diagnosed at late stage and has high rate of postoperative metastasis and recurrence, thus the mortality rate is high [4]. Exploring the mechanisms of HCC metastasis and recurrence is critical to the development of effective therapeutic approaches.

HCC progression is associated with many factors, such as chronic alcohol consumption [5–7]. The International Agency for Research on Cancer (IARC) lists alcohol as the cause of various cancers (including liver cancer) [8]. Alcohol consumption accounts for 3.6% of cancer cases and 3.5% of cancer deaths [9]. Recent cohort studies show a positive relationship between the dosage of alcohol consumption and progression of liver cancer [10]. However, the underlying mechanism still needs to be fully studied.

Cancer stem cells (CSC) have self-renewal and differentiation capabilities [11], which are closely related to tumor metastasis and recurrence [12]. Liver cancer stem cells (LCSCs) are responsible for HCC relapse, metastasis, and chemoresistance [13]. Markers of LCSCs are epithelial cell adhesion molecule (EpCAM, CD326), CD133, CD90, CD44, CD13 [11, 14]. EPCAM positive HCC cells display stem cells ability [15]. Wnt-*β*-catenin signaling pathway is activated in EPCAM positive HCC cells [16, 17]. CD133 positive HCC cells also have stem cells capacity [18]. CD133 is an important prognostic indicator for HCC patients [19]. CD90^+^ HCC cells isolated from HCC cells display stronger tumorigenicity [20]. CD133 and CD90 are related to the expression of fetal liver cell markers, tumor occurrence and drug resistance [21, 22]. CD44 is also a marker of LCSCs and is involved in maintaining the stemness of LCSCs [23]. CD133^+^ CD13^+^ HCC cells have strong stem cell ability [24] and drug resistance [25]. Inhibition of CD13 suppresses the self-renewal ability of LCSCs [26]. However, EpCAM and CD133 are the most common and conclusive markers of LCSCs. In this study, we used EPCAM and CD133 as markers of LCSCs.

microRNA (miRNA) deregulation is frequently occurred in cancers. miR-22-3p promotes hematopoietic stem cells self-renewal ability by targeting TET2 [27]. miR-22-3p also promotes breast cancer cells stemness through suppressing TET1 and TET2 [28]. TET2 is a methyltransferase to demethylate DNA [29]. DNA methylation is closely related to tumorigenesis [30]. TET2 is closely related to the self-renewal and differentiation of CSCs [31, 32]. However, the function of TET2 in HCC and LCSCs remains unknown. Our study attempts to explore the role of TET2 in the malignant progression of HCC. Recent studies indicate that CSCs are related to epithelial-mesenchymal transitions (EMT), a process that makes cancer cells more aggressive [33]. However, the relationship between LCSCs and the occurrence of EMT is unclear. Our research will also explore LCSC-enhanced stemness on HCC cells metastasis. Wnt, Noth, and Hh signaling pathways participated in the regulation of cancer stem cells [34–37]. Wnt-*β*-catenin is associated with cell-cell adhesion and EMT process [38]. *β*-catenin aberrant activation is observed in 20-35% cases of HCC [39]. Wnt-*β*-catenin-mediated enrichment of EpCAM positive CSCs promotes drug resistance in HCC [40]. We will further study the role of *β*-catenin and its relationship with miR-22-3p.

In this study, we investigated the function of miR-22-3p and TET2 in alcohol-promoted HCC progression and the underlying mechanisms through evaluating clinical data and experimental models.

## RESULTS

### Chronic alcohol exposure promotes hepatocellular carcinoma metastasis

**Figure 1:**
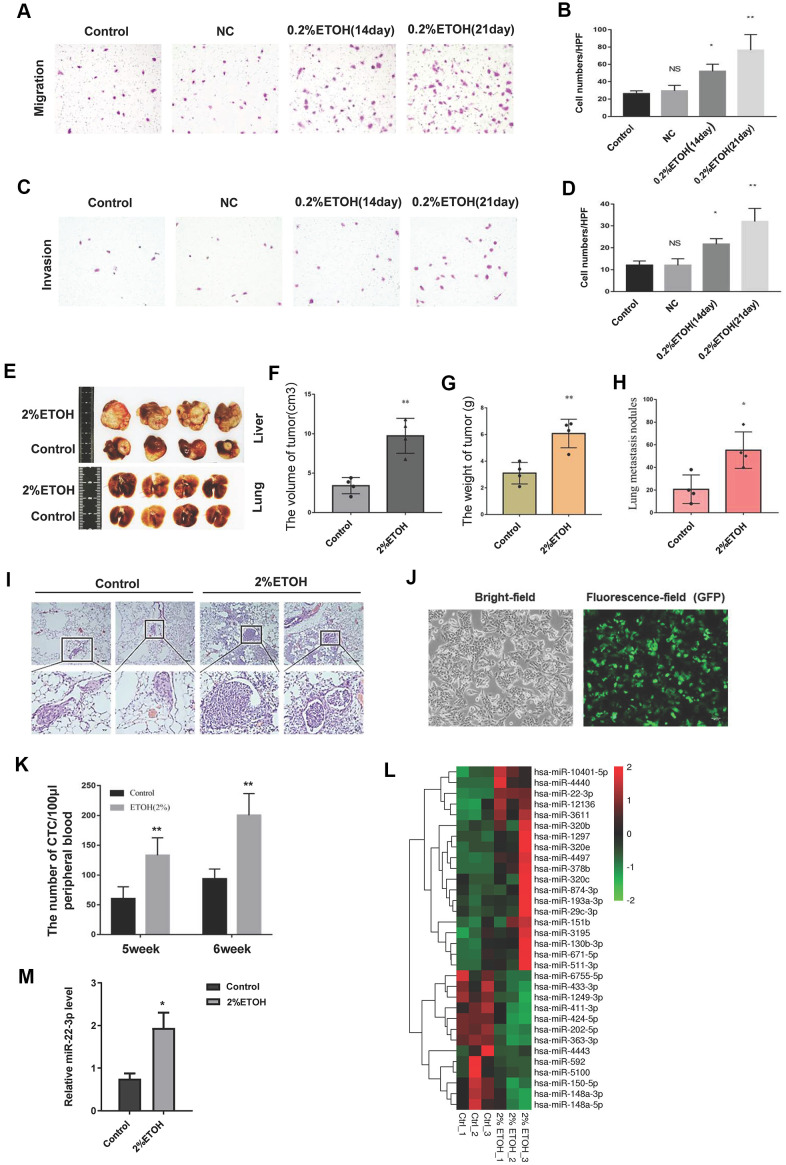
**Chronic alcohol exposure promotes HCC metastasis.** (**A**) A representative image showing the migration of control, negative control (NC) and chronic alcohol-induced HCC cells. (**B**) The migrated cells were quantified and shown in column graph. *P< 0.05, **P< 0.01, n= 3. (**C**) A representative image showing the invasion of control, NC and chronic alcohol-induced HCC cells. (**D**) The invaded cells were quantified and shown in column graph. *P< 0.05, **P< 0.01, n= 3. (**E**) Typical anatomical images of orthotopic liver tumors and lung metastasis nodules from the control group and alcohol drinking nude mice. (**F**) The volume of orthotopic liver tumors from the control group and alcohol drinking nude mice. Each group consisted of four mice. **P< 0.01. (**G**) The weight of orthotopic liver tumors from the control group and alcohol drinking nude mice. Each group consisted of four mice. **P < 0.01. (**H**) The number of lung metastasis nodules from the control group and alcohol drinking nude mice was quantified. Each group consisted of four mice. *P< 0.05. (**I**) HE images of lung metastasis nodules from the control group and alcohol drinking nude mice. (**J**) The bright-field and fluorescence-field images of GFP-labelled HCC-LM3 cells. (**K**) The number of CTCs in peripheral blood of the orthotopic tumor model mice. **P< 0.01, n= 4. (**L**) microRNA-sequencing results of liver tumor tissues from the control group and alcohol drinking nude mice. (**M**) The expression level of miR-22-3p in tumors from the control group and alcohol drinking nude mice. Each group consisted of four mice. *P< 0.05.

We first studied the effect of alcohol on HCC metastasis *in vitro*. HCC-LM3 cells were divided into three groups, the control group was cultured routinely, and the alcohol exposure group was treated with 0.2% v/v alcohol for 14 days or 21 days. Alcohol exposure promoted the HCC-LM3 cells migration and invasion (Figure 1A–1D). *In vivo*, we constructed an orthotopic tumor model by direct injecting HCC-LM3 cells into the liver of BALB/C nude mice. As shown in Figure 1E–1G, chronic alcohol consumption increased the formation of liver tumors in BALB/C nude mice. Alcohol also enhanced the lung metastasis nodules formation (Figure 1E, 1H, 1I). Additionally, we found that alcohol exposure increased the number of circulating GFP-labeled HCC-LM3 cells in peripheral blood of the orthotopic tumor model mice (Figure 1J, 1K). These results indicate that chronic alcohol exposure promotes HCC progression, including metastasis and pulmonary nodules formation.

We performed microRNA-sequencing study in the liver tumor tissues from the control and alcohol drinking nude mice. As shown in Figure 1L, many microRNAs were up-regulated in alcohol group. Interestingly, miR-22-3p was particularly high in alcohol group (Figure 1M). miR-22-3p acts as an oncogene in breast cancer, promotes breast cancer cells metastasis and stemness [28]. Besides, miR-22-3p is up regulated in alcohol-induced MCF-7 cells (GEO accession GSE72012; Gelfand R et al., 2017). However, miR-22-3p function in HCC and LCSCs is still unclear. Thus, we thought to investigate miR-22-3p function in alcohol promoted HCC progression.

### miR-22-3p is highly expressed in HCC and alcohol enhances this effect

**Figure 2:**
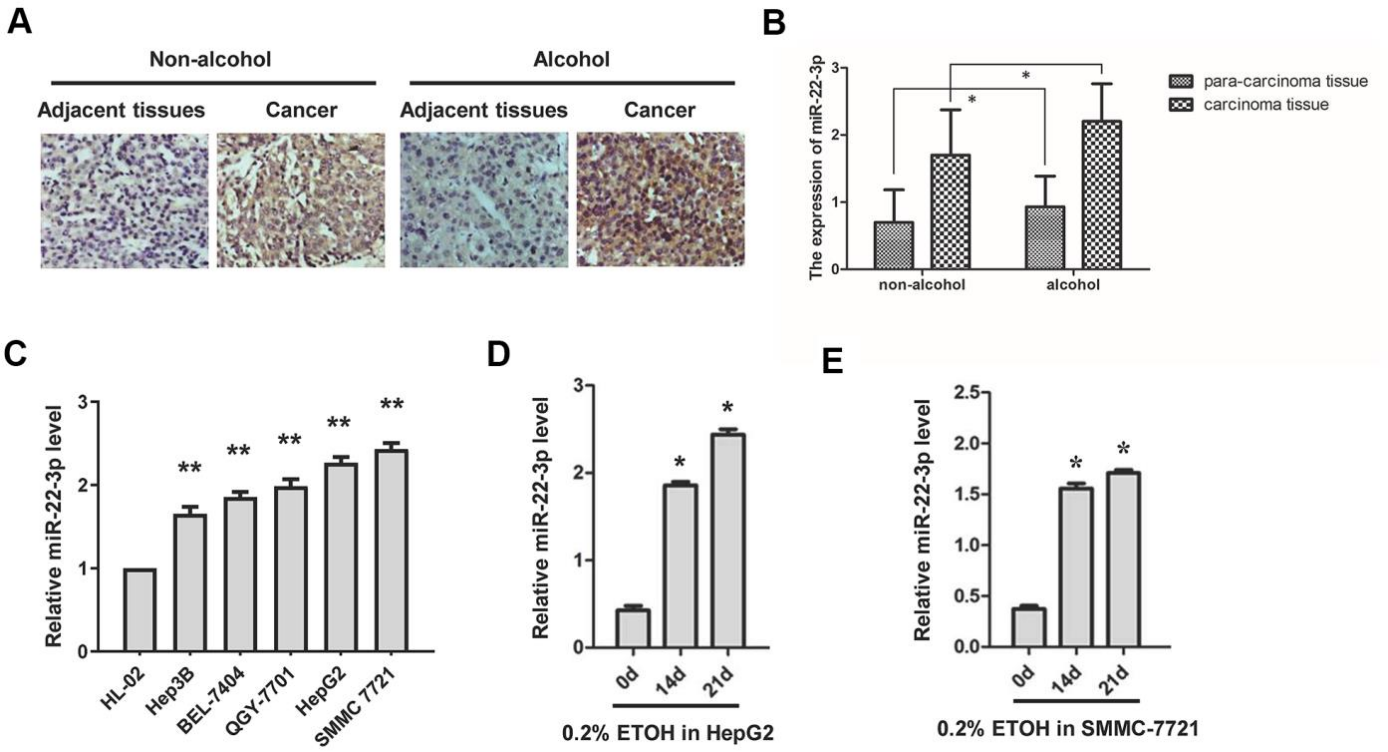
**Expression of miR-22-3p in HCC tissues and cell lines with or without alcohol exposure.** (**A**) ISH detection of miR-22-3p in adjacent and cancerous tissues of HCC patients with or without alcohol consumption history. Original magnification, ×400. (**B**) The miR-22-3p expression level in (**A**) is quantified and shown in column graph. n= 25. (**C**) Relative miR-22-3p level in HCC cells and normal liver cells. **P< 0.01, n= 3. (**D**) Relative expression level of miR-22-3p in HepG2 cells treated with alcohol for 0, 14 or 21 days. (**E**) Relative expression level of miR-22-3p in SMMC-7721 cells treated with alcohol for 0, 14 or 21 days.

To evaluate the function of miR-22-3p in alcohol-promoted HCC, we first performed ISH on HCC tissues and para-cancer tissues of clinical patients with or without alcohol consumption. miR-22-3p was highly expressed in HCC tissues than their corresponding adjacent tissues, and alcohol exposure increased miR-22-3p expression both in HCC tissues and their corresponding adjacent tissues (Figure 2A, 2B). We then examined miR-22-3p expression in five human HCC cell lines (Hep3B, BEL-7404, QGY-7701, HepG2 and SMMC-7721) and normal liver cell HL-02. Compared with normal liver cells, miR-22-3p was highly expressed in HCC cell lines (Figure 2C). We also found that alcohol exposure drastically upregulated miR-22-3p in HepG2 and SMMC-7721 cells (Figure 2D, 2E).

### miR-22-3p promotes HCC stemness and metastasis

**Figure 3:**
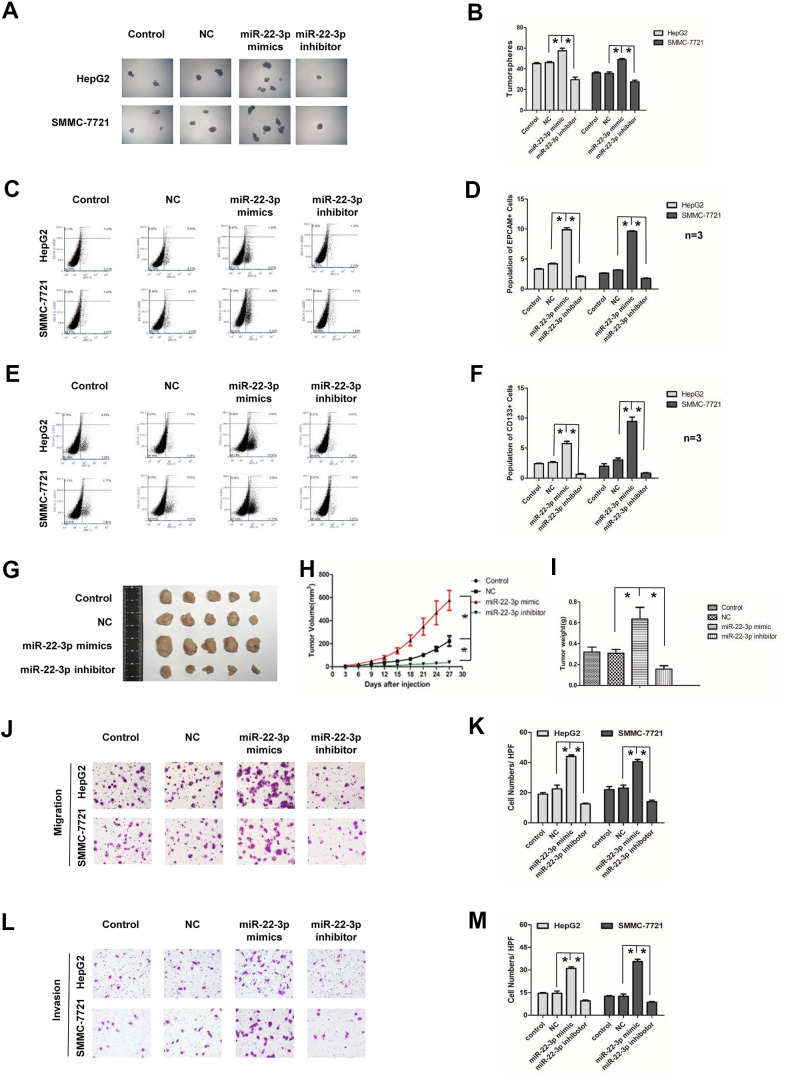
**Effects of miR-22-3p on stemness and metastasis of HCC cells.** (**A**) Tumorspheres formation ability of HCC cells in control, negative-control, miR-22-3p mimic and inhibitor groups. (**B**) The tumorspheres were quantified. *P<0.05. (**C**, **D**) Population of EPCAM- positive HCC cells in control, negative-control, miR-22-3p mimic and inhibitor groups. *P<0.05. (**E**, **F**) Population of CD133- positive HCC cells in control, negative-control, miR-22-3p mimic and inhibitor groups. *P<0.05. (**G**) The representative images of tumors taken from athymic mice inoculated with SMMC-7721 cells in control, negative-control, miR-22-3p mimic and inhibitor groups are shown. (**H**) The growth of tumor was calculated. Each group consisted of five mice. *P< 0.05. (**I**) The tumor weight was quantified. Each group consisted of five mice. *P< 0.05. (**J**) Representative image showing the migration of HCC cells in control, negative-control, miR-22-3p mimic and inhibitor groups. (**K**) The migrated cells were quantified. *P< 0.05, n= 3. (**L**) Representative image showing the invasion of HCC cells in control, negative-control, miR-22-3p mimic and inhibitor groups. (**M**) The invaded cells were quantified. *P< 0.05, n= 3.

We used both gain-of function and loss-of function approaches to study the function of miR-22-3p in HCC. HCC cells were infected with negative control, miR-22-3p mimic or miR-22-3p inhibitor lentivirus. The success of transfection was displayed by GFP fluorescence and miR-22-3p expression was confirmed by real-time q-PCR (Supplementary Figure 1A–1D). In the tumorspheres culture assay, the size and number of tumorspheres in miR-22-3p-overexpressed HCC cells were greater than those of the control group; while the miR-22-3p inhibition showed an opposite effect (Figure 3A, 3B). To further investigate the effect of miR-22-3p on the stemness of liver cancer stem cells, we detected the positive populations of EPCAM and CD133 in HCC cells. Overexpression of miR-22-3p increased the number of EPCAM and CD133 positive cells in HCC cells. However, miR-22-3p inhibition reduces the population (Figure 3C–3F). In animal study, SMMC-7721 cells stably infected with negative control, miR-22-3p mimic and miR-22-3p inhibitor lentivirus were subcutaneously injected into BALB/C nude mice. Tumors in miR-22-3p mimic group grew faster and bigger than the control group; while the miR-22-3p inhibitor group grew slower and smaller than the control group (Figure 3G, 3H). The weight of tumor in miR-22-3p mimic group was significantly heavier compared with the control group; while the miR-22-3p inhibitor group was lighter than the control group (Figure 3I, p < 0.01).

Further, we detected the function of miR-22-3p on HCC metastasis. In HCC cell lines, miR-22-3p mimic group showed stronger metastasis and invasion potential than the control group; while the miR-22-3p inhibitor group have less metastasis and invasion ability than the control group (Figure 3J–3M). The above results indicated that miR-22-3p enhanced stemness and metastasis of HCC cells.

### TET2 is a direct downstream target of miR-22-3p

We further sought to determine the potential downstream target of miR-22-3p. The online bioinformatics software Target Scan predicted that a complementary site of miR-22-3p was identified in the 3’-UTR of TET2 mRNA (Supplementary Figure 2A). The relationship between TET2 and miR-22-3p was confirmed by dual-luciferase reporter gene system. TET2 wild-type or mutant 3'-UTR contains a putative miR-22-3p binding site, which is cloned into the downstream region of the Photinus pyralis/Renilla double-Luciferase reporter gene in the psi-CHECK2 reporter vector. HL-02 cells were co-infected with wild-type 3'-UTR reporter vector and miR-22-3p mimic, showing that luciferase activity was significantly reduced, while the luciferase activity in cells infected with mutant-3'-UTR vector was not affected (Supplementary Figure 2B).

In HepG2 cells, miR-22-3p is highly expressed (Supplementary Figure 2C). miR-22-3p inhibitor and wild-type 3'-UTR reporter vector/ mutant-3'-UTR vector were co-transfected into HepG2 cells. luciferase activity in cells transfected with the wild-type 3'-UTR reporter vector was significantly increased, while Luciferase activity in cells transfected with the mutant-3'-UTR vector was not changed (Supplementary Figure 2C). miR-22-3p mimic or its negative control were transfected into HL-02 cells. TET2 protein level was down-regulated in miR-22-3p mimic group (Supplementary Figure 2D–2F). Besides, miR-22-3p inhibitor or its negative control were transfected into HepG2 cells. TET2 protein level was up-regulated in miR-22-3p inhibitor group (Supplementary Figure 2G–2I). In summary, miR-22-3p negatively regulate TET2, which is a direct downstream target of miR-22-3p.

### TET2 expression is downregulated in HCC and alcohol enhances this effect

**Figure 4:**
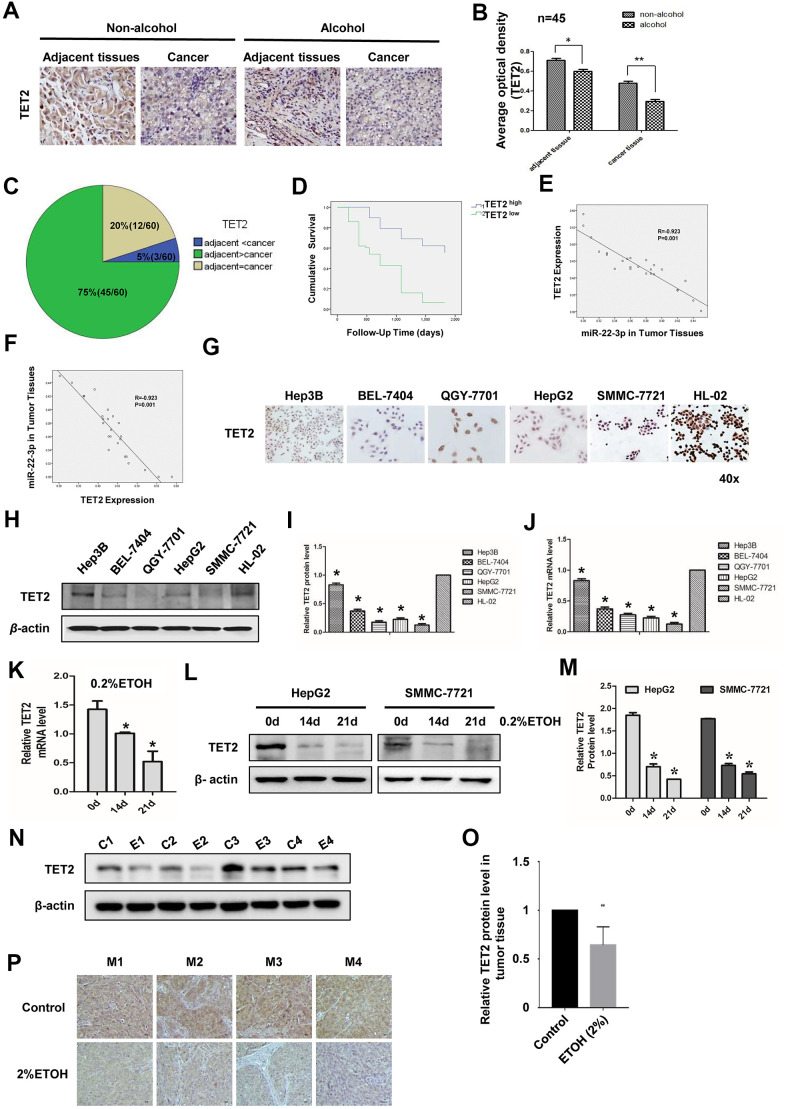
**Expression of TET2 in HCC tissues and cell lines with or without alcohol exposure.** (**A**) IHC detection of TET2 protein in adjacent and cancerous tissues of HCC patients with or without alcohol consumption history. Original magnification, ×400. (**B**) B is the quantification of A, n=45. (**C**) Statistical analysis of TET2 protein expression in adjacent and cancerous tissues of 60 HCC specimens. (**D**) Survival curve of HCC patients in TET2 high expression and low expression group. (**E**) Co-relationship analysis of miR-22-3p and TET2. R= -0.923, *P*= 0.001. (**F**) Co-relationship analysis of TET2 and miR-22-3p. R= -0.923, *P*= 0.001. (**G**) Protein level of TET2 in HCC cells and normal liver cells by cell IHC. Hep3B, BEL-7404, QGY-7701, HepG2 and SMMC-7721 are hepatocellular carcinoma cells while HL-02 is normal liver cells. (**H**) Protein level of TET2 in HCC cells and normal liver cells by Western blotting. (**I**) I is the quantification of H. (**J**) The relative mRNA level of TET2 in HCC cells and normal liver cells. (**K**) The mRNA expression levels of TET2 in HepG2 cells treated with 0.2% v/v alcohol for 0, 14, or 21 days. (**L**) The protein level of TET2 in HepG2 or SMMC-7721 cells treated with 0.2% v/v alcohol for 0, 14, or 21 days. (**M**) The protein level was quantified and shown in column graph. *P< 0.05, n = 3. (**N**) The protein level of TET2 in orthotopic liver tumors from the control group and alcohol drinking nude mice was analyzed by WB. Each group consisted of four mice. (**O**) The protein level of TET2 in N was quantified and shown in column graph. *P< 0.05, n = 4. (**P**) The protein level of TET2 in orthotopic liver tumors from the control group and alcohol drinking nude mice was analyzed by IHC. Each group consisted of four mice.

We then studied the potential role of TET2 in HCC. TET2 was down-regulated in HCC tissues (Figure 4A, 4B). Alcohol exposure decreased TET2 level not only in adjacent tissues but also in tumor tissues (Figure 4A, 4B). We analyzed 60 pairs of HCC specimen. TET2 was downregulated in 75% HCC specimen and upregulated in 20% HCC specimen compared with para-cancer tissues. TET2 levels was unchanged in 5% HCC specimen compared with para-cancer tissues (Figure 4C). Besides, TET2 level is negatively correlated with the survival rate of HCC patients (Figure 4D). Correlation analysis of miR-22-3p and TET2 in HCC tissues showed that miR-22-3p was inversely correlated with TET2 (Figure 4E, 4F).

Compared with normal liver cells, TET2 decreased in HCC cell lines both at protein level (Figure 4G–4I) and mRNA level (Figure 4J). Furthermore, we investigated alcohol exposure on the expression level of TET2 in HCC cells. Alcohol treatment decreased the expression level of TET2 (Figure 4K–4M). In addition, the level of TET2 in tumor tissues formed by HCC-LM3 cells was significantly inhibited by alcohol exposure *in vivo* (Figure 4N–4P). These results demonstrated that TET2 was downregulated in HCC and alcohol enhanced this effect.

### TET2 suppresses HCC stemness and metastasis

**Figure 5:**
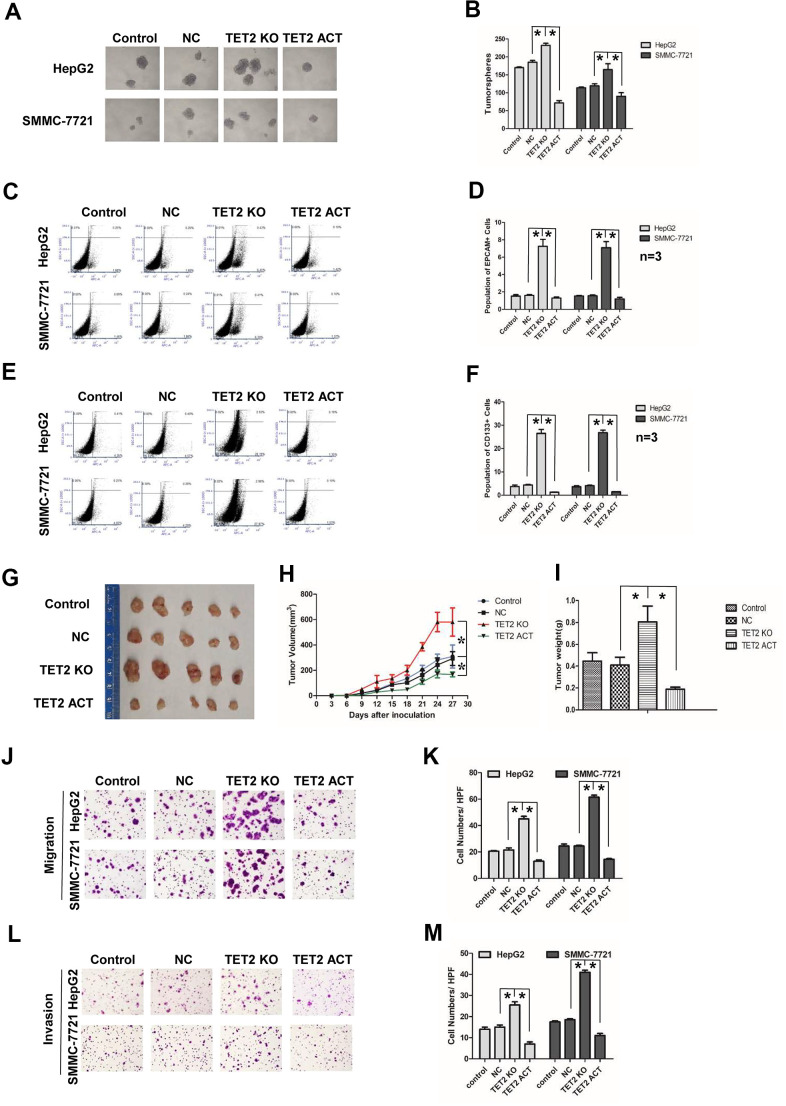
**Effects of TET2 on stemness and metastasis of HCC cells.** (**A**) Tumorspheres formation ability of HCC cells in control, negative-control, TET2 Knockout (KO) and TET2 Activation (ACT) groups. (**B**) The tumorspheres were quantified. *P< 0.05. (**C**, **D**) Population of EPCAM- positive HCC cells in control, negative-control, TET2 Knockout (KO) and TET2 Activation (ACT) groups. *P< 0.05. (**E**, **F**) Population of CD133-positive HCC cells in control, negative-control, TET2 Knockout (KO) and TET2 Activation (ACT) groups. *P< 0.05. (**G**) The representative images of tumors taken from athymic mice inoculated with SMMC-7721 cells in control, negative-control, TET2 Knockout (KO) and TET2 Activation (ACT) groups are shown. (**H**) The growth of tumor was calculated. Each group consisted of five mice. *P< 0.05. (**I**) The tumor weight was quantified. Each group consisted of five mice. *P< 0.05. (**J**) Representative images showing the migration of HCC cells in control, negative-control, TET2 Knockout (KO) and TET2 Activation (ACT) groups. (**K**) The migrated cells were quantified. *P< 0.05, n= 3. (**L**) Representative image showing the invasion of HCC cells in control, negative-control, TET2 Knockout (KO) and TET2 Activation (ACT) groups. (**M**) The invaded cells were quantified. *P< 0.05, n= 3.

To further study the function of TET2, we established stable HCC cell lines either overexpressing or down-regulating TET2 by transfecting TET2 CRISPR/Cas9 Knockout (KO) or TET2 CRISPR/Cas9 Activation (ACT) plasmids. The transfection efficiency was confirmed by western blotting (Supplementary Figure 3A–3D). LCSCs have ability of self-renewal and high ability of tumorigenesis and metastasis [14, 16]. We first examined the effect of TET2 on HCC stemness. In the tumorspheres culture assay, Knockout TET2 promoted tumorspheres growth, while the TET2 ACT HCC cells showed an opposite result (Figure 5A, 5B). Furtherly, we analyzed EPCAM and CD133 positive cells in TET2 KO/ACT HCC cells. We found that the population of EPCAM+ and CD133+ cells in the TET2 KO group increased, while activation of TET2 showed a decrease tendency (Figure 5C–5F). In the *in vivo* study, SMMC-7721 cells stably transfected with TET2 KO and TET2 ACT plasmid were subcutaneously injected into BALB/C nude mice. Notably, compared with the control group, tumors of TET2 KO group grew much faster and bigger while TET2 ACT group showed opposite results (Figure 5G, 5H). Compared with the control group, the weight of tumor in TET2 KO group was much heavier while the TET2 ACT group showed opposite result (Figure 5I, *P*< 0.01).

We then examined the effect of TET2 on HCC metastasis. TET2 KO cells showed more metastasis and invasion potential compared to control cells, while the TET2 ACT cells displayed less metastasis and invasion (Figure 5J–5M). Epithelial to mesenchymal transition (EMT) occurs during tumor cells metastasis [41]. In EMT process, epithelioid-like markers, E-cadherin are down-regulated, while mesenchymal-like markers, such as N-cadherin and Vimentin are up-regulated [42]. In TET2 KO HCC cells, E-cadherin was down-regulated, while N-cadherin and Vimentin were up-regulated (Supplementary Figure 3E–3H). However, activation of TET2 partially reversed the EMT process, especially the N-cadherin expression (Supplementary Figure 3E–3H). Together, these results indicate that TET2 suppresses HCC stemness and metastasis.

### miR-22-3p promoted stemness and metastasis of HCC via targeting TET2

**Figure 6:**
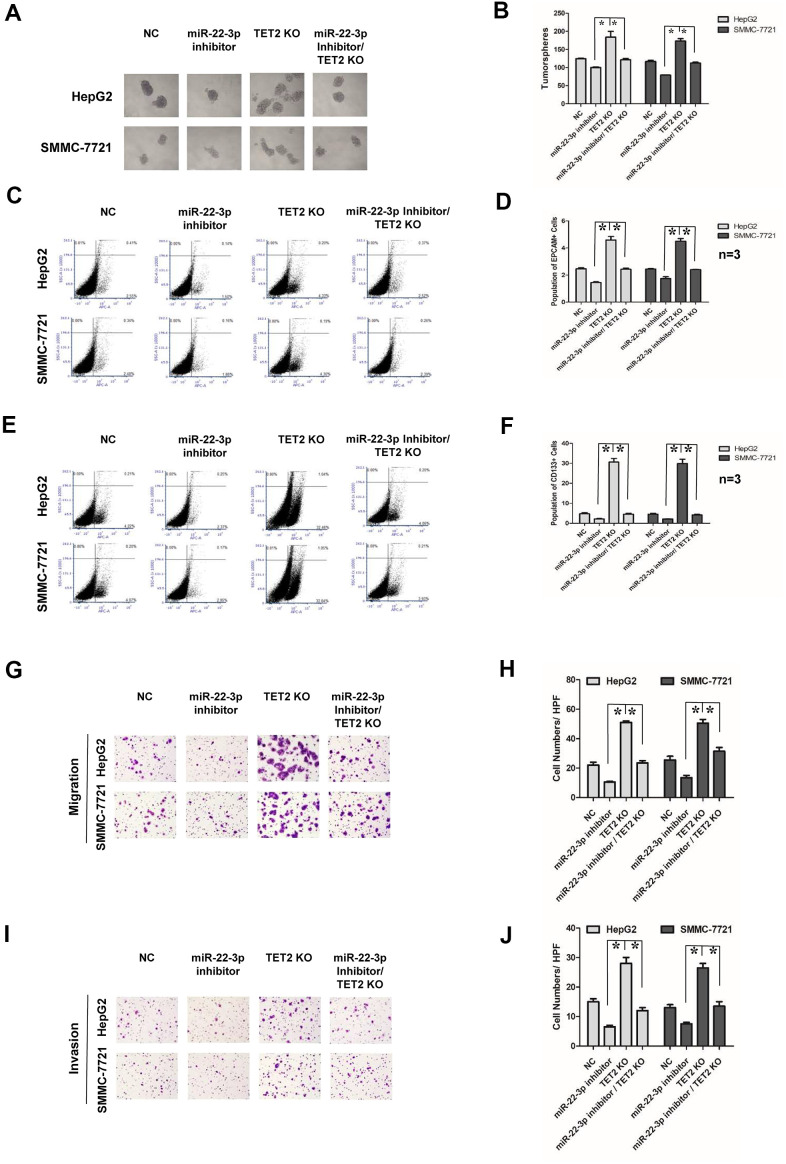
**Effects of miR-22-3p and TET2 on stemness and metastasis of HCC cells.** (**A**) Tumorspheres formation ability of HCC cells in negative-control, miR-22-3p inhibitor, TET2 KO and miR-22-3p inhibitor/TET2 KO groups. (**B**) The tumorspheres were quantified. *P<0.05. (**C**, **D**) Population of EPCAM-positive HCC cells in negative-control, miR-22-3p inhibitor, TET2 KO and miR-22-3p inhibitor/TET2 KO groups. *P<0.05. (**E**, **F**) Population of CD133- positive HCC cells in negative-control, miR-22-3p inhibitor, TET2 KO and miR-22-3p inhibitor/TET2 KO groups. *P<0.05. (**G**) Representative image showing the migration of HCC cells in control, negative-control, miR-22-3p inhibitor, TET2 KO and miR-22-3p inhibitor/TET2 KO groups. (**H**) The migrated cells were quantified as described in the Materials and Methods. *P< 0.05, n= 3. (**I**) Representative image showing the invasion of HCC cells in negative-control, miR-22-3p inhibitor, TET2 KO and miR-22-3p inhibitor/TET2 KO groups. (**J**) The invaded cells were quantified as described in the Materials and Methods. *P< 0.05, n= 3.

We next investigated whether miR-22-3p-mediated suppression of stemness and metastasis requires TET2. miR-22-3p inhibitor and TET2 KO plasmid were co-expressed in HCC cells. The success of co-transfection was demonstrated by both GFP and RFP fluorescence, and the transfection efficiency was confirmed by Western blotting (Supplementary Figure 4A–4F). As shown in tumorspheres culture assay (Figure 6A, 6B), miR-22-3p inhibitor and TET2 KO co-expression reversed the effects of TET2 KO on HCC cells. miR-22-3p inhibitor and TET2 KO co-expression also inhibited the effect of TET2 KO on stem cells population of HCC cells (Figure 6C–6F). The similar effect of the miR-22-3p inhibitor and TET2 KO co-expression on metastasis and invasion was observed (Figure 6G–6J). In conclusion, the above results indicated that miR-22-3p promotes stemness and metastasis of HCC through TET2.

### *β*-catenin/miR-22-3p/TET2 axis participated in alcohol-promoted HCC malignancy

**Figure 7:**
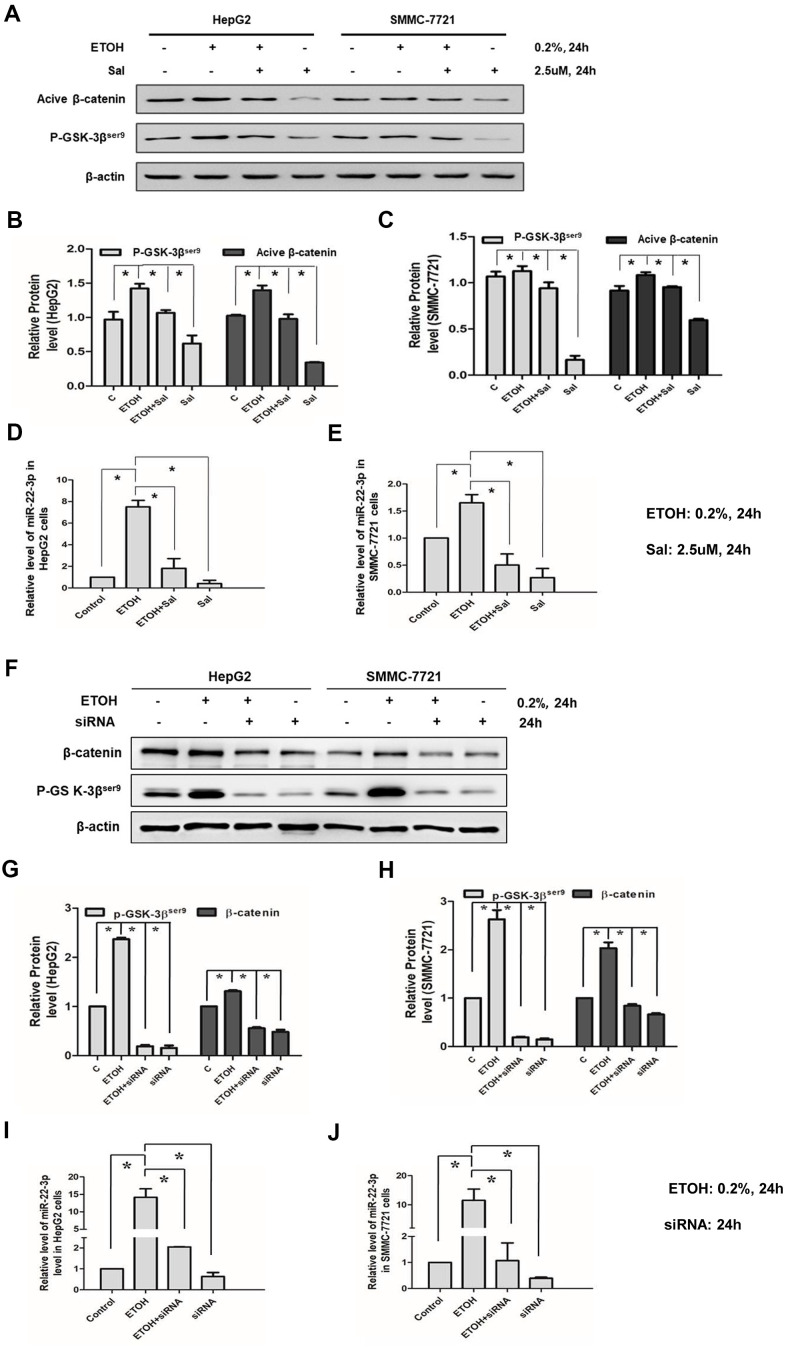
**Effects of salinomycin/ *β*-catenin siRNA and alcohol exposure on *β*-catenin signaling and miR-22-3p.** (**A**) The effect of salinomycin and alcohol exposure on *β*-catenin signaling in HepG2 or SMMC-7721 cells. (**B**) The protein levels of p-GSK-3*β*^ser9^ and active *β*-catenin in HepG2 cells were qualified and shown in column graph. *P< 0.05. (**C**) The protein levels of p-GSK-3*β*^ser9^ and active *β*-catenin in SMMC-7721 cells were qualified and shown in column graph. *P< 0.05. (**D**) The effect of salinomycin and alcohol exposure on miR-22-3p in HepG2 cells. *P< 0.05. (**E**) The effect of salinomycin and alcohol exposure on miR-22-3p in SMMC-7721 cells. *P< 0.05. (**F**) The effect of *β*-catenin siRNA and alcohol exposure on *β*-catenin signaling in HepG2 or SMMC-7721 cells. (**G**) The protein levels of p-GSK-3*β*^ser9^ and active *β*-catenin in HepG2 cells were qualified and shown in column graph. *P< 0.05. (**H**) The protein levels of p-GSK-3*β*^ser9^ and active *β*-catenin in SMMC-7721 cells were qualified and shown in column graph. *P< 0.05. (**I**) The effect of *β*-catenin siRNA and alcohol exposure on miR-22-3p in HepG2 cells. *P< 0.05. (**J**) The effect of *β*-catenin siRNA and alcohol exposure on miR-22-3p in SMMC-7721 cells. *P< 0.05.

Wnt-*β*-catenin is a regulator of CSCs [34] and EMT [38] and activation of the Wnt-*β*-catenin increases EPCAM positive HCC cells [16, 17]. In the previous study, we found that alcohol exposure activated *β*-catenin signal and lead to EMT of HCC cells [43]. In the current study, we sought to determine whether *β*-catenin correlated with miR-22-3p in alcohol-promoted HCC progression. Salinomycin treatment (2.5 μM, 24 hours) had been demonstrated could inhibit Wnt-*β*-catenin signaling in HCC cells in our previous study [43]. We showed here that salinomycin (2.5 μM, 24 hours) down-regulated miR-22-3p; salinomycin also inhibited alcohol exposure increased miR-22-3p expression (Figure 7A–7E). Besides, we used *β*-catenin siRNA to specifically knockdown *β*-catenin. We treated HCC cells with *β*-catenin siRNA for 24 hours, miR-22-3p expression was decreased when *β*-catenin (Active, non-phosphorylated) was inhibited. *β*-catenin siRNA also reversed the effect of alcohol exposure on miR-22-3p (Figure 7F–7J). In conclusion, these results demonstrated that *β*-catenin/miR-22-3p/TET2 axis participates in alcohol-promoted HCC malignancy.

## DISCUSSION

The increased risk of multiple malignancies is related to alcohol abuse, leading to poor prognosis for cancer patients [44, 45]. Our previous study showed that alcohol exposure promoted colorectal carcinoma malignant progression through activation of autophagy [46]. Recently, we demonstrated that alcohol enhanced progression, metastasis and stemness of hepatocellular carcinoma via EMT and NF-κB pathways [43, 47]. However, the exact mechanisms for 25% of liver cancer are unclear [48]. In this study, we used an innovative approach employing the orthotopic liver tumor model to investigate the effect of alcohol on HCC progression *in vivo*. We found that alcohol exposure promoted HCC progression, enhancing metastasis and pulmonary nodules formation. Furthermore, in this process, miR-22-3p played a critical role via targeting TET2.

Patients with HCC have a poor prognosis in developing countries and the 5-year survival rate is only about 5% [49]. This is partially due to that early HCC patients do not have obvious clinical symptoms and lack of markers for the diagnosis of aggressive HCC. Due to poor understanding of underlying molecular mechanisms, there is not effective and specific treatment for HCC. microRNAs have been proposed to be involved in tumorigenesis and progression [50]. miR-22-3p is implicated in prostate cancer and breast cancer. In human prostate cancer, miR-22-3p promotes prostate cancer progression through targeting the tumor suppressor gene PTEN [51]. In human breast cancer, miR-22-3p promotes breast cancer stem cells differentiation and breast cancer cells metastasis [28]. Here, we showed that miR-22-3p promotes tumorspheres formation of HCC cells, increases stem cell proportion and tumorigenicity in nude mice, while miR-22-3p inhibitor reverses these processes. miR-22-3p inhibition blocks HCC cells metastasis. In HCC, miR-22-3p is highly expressed and inversely correlated with TET2. These results suggest that miR-22-3p may be used as a diagnostic and prognostic index for HCC.

In addition, we showed that miR-22-3p negatively regulate TET2. It has been reported that the absence and mutation of TET2 occurred in hematic tumors [27], and the lack of TET2 increased the stemness and metastasis of breast cancer cells [28]. Here, we demonstrated that TET2 down-regulation resulted in malignant progression of HCC cells. This was supported by the increase of tumorspheres and proportion of LCSCs *in vitro*, and rapid growth of nude mice tumors *in vivo*. In TET2 gene knockout HCC cells, a significant increase in cell metastasis was observed. In clinical HCC patients, expression of TET2 was inversely associated with the survival rate. Therefore, low level of TET2 expression may be accountable for the high metastasis rate, recurrence, and poor clinical prognosis in HCC patients. As a result, TET2 may be used for early screening and diagnosis of prognosis in HCC patients. Additionally, we showed that activating TET2 inhibited the formation of tumorspheres, and decreased LCSCs population. Animal studies confirmed that activation of TET2 inhibited the growth of HCC cells. TET2 activation also blocked HCC cells metastasis. Therefore, TET2 may be used as a new therapeutic target for HCC metastasis and recurrence.

In cancer metastasis, cells usually underwent EMT process. We showed that TET2 inhibition increased the expression of EMT markers in HCC, while activation of TET2 inhibited this effect. This is consistent with that activation of TET2 blocked HCC cells metastasis. This result suggests that TET2 is a regulator in the EMT process of HCC cells. EMT is regulated by a cascade of cell signaling network [52]. Our previous study showed that chronic alcohol exposure increased the stemness and metastasis of HCC and promoted EMT process by activating *β*-catenin signaling pathway [43]. In this study, we confirmed that *β*-catenin was an upstream activator of miR-22-3p and involved in alcohol-promoted HCC progression. In summary, we demonstrated that *β*-catenin/miR-22-3p/TET2 axis is involved in alcohol-promoted HCC stemness and metastasis (Supplementary Figure 5). miR-22-3p and TET2 may serve as markers for HCC prognosis and potential clinic targets of HCC.

## MATERIALS AND METHODS

### Drugs and alcohol exposure

Salinomycin and GSK-3 inhibitor SB-216763 were ordered from Sigma. β-catenin siRNA was ordered from Santa Cruz. Alcohol-induced HCC cells were treated with 0.2% v/v alcohol as our previous study [47].

### Migration and invasion assay

In migration assay, the culture medium contained 10% serum in the bottom of the transwell chambers (BD Biotechnology). Control or experimental group HCC cells (0.2 million) were re-suspended in serum-free medium and plated in the upper chamber. Cells migrated through the filter were stained with 0.5% crystal violet containing paraformaldehyde for 1 hours. The microscope (Zeiss, Germany) was used for photographed. In invasion assay, matrigel (BD Biosciences, USA) were added to the upper chamber.

### Orthotopic transplantation tumor model

GFP tagged HCCLM3 cells were harvested and resuspended in PBS (2.5 million cells per mL), which was then diluted by adding equal volume matrigel. 40μL cell suspension was orthotopically transplanted into livers of anesthetized BALB/C nude mice. Seven days later, the mice returned to normal and were fed with water or alcohol (2%). Peripheral blood samples were taken at the 5th and 6th weeks after water or alcohol drinking for circulating tumor cells (CTC) counting. The experiment was ended after 7 weeks. The livers and lungs of the mice were taken for follow-up experiments as indicated.

### Clinical data and specimen

HCC clinical samples used in this study were confirmed by pathology and obtained from the First Affiliated Hospital of Anhui Medical University. All associated experiments were approved by the Human Research Committee of Anhui Medical University.

### Lentivirus infection assay

Lentivirus miR-22-3p inhibitor/mimic and their negative control were bought from Genechem (Shanghai, China). Lentivirus transducing units for HCC cells is 1×107. Stable miR-22-3p inhibitor/mimic and their negative control HCC cells were selected by puromycin.

### CRISPR/Cas9 transfection assay

TET2 CRISPR/Cas9 Knock-out (KO) plasmid and TET2 HDR plasmid were co-transfected into HCC cells to knock out TET2 gene expression. TET2 CRISPR/Cas9 KO plasmid incorporates a GFP gene. TET2 HDR plasmid incorporates an RFP gene and a puromycin resistance. TET2 CRISPR/Cas9 Activation (ACT) plasmid was transfected into HCC cells to upregulate TET2 gene expression in HCC cells. TET2 ACT plasmid incorporates a puromycin resistance gene. TET2 CRISPR/Cas9 Knock-out (KO) plasmid, TET2 HDR plasmid and TET2 CRISPR/Cas9 Activation (ACT) plasmid were bought from Santa Cruz Biotechnology (USA). The plasmid transfection is according to the user’s protocol.

### Dual luciferase reporter gene assay

miR-22-3p mimic/NC and TET2 WT plasmid/ TET2 MUT plasmid were co-transfected into HL-02 cells. miR-22-3p inhibitor/NC and TET2 WT plasmid/ TET2 MUT plasmid were co-transfected into HepG2 cells. After 48 hours transfection, Dual Luciferase Report Assay System (Promega USA) was used to detect firefly and renilla luciferase activities.

### Tumorspheres culture assay

HCC cells were counted at a concentration of 1×10^4^ cells per well and then cultured in stem cell culture medium. Furtherly, fresh medium (200 μL) was added every 2-3 days. After 14 days, tumorspheres were then photographed and counted.

### Flow cytometry assay

1×106/ml HCC cells were collected in 100ul PBS. Anti-EPCAM (eBioscience, USA) and Anti-CD133 (eBioscience, USA) were added into HCC cells suspension and incubated for 30 minutes on ice. Then wash the cell pellet with PBS. Resuspend cell pellet with fresh PBS and analysis with flow cytometry.

### Tumor xenograft assay

1×106/100ul control and experiment HCC cells (SMCC-7721) in PBS were inoculated subcutaneously into the armpit of BALB/C nude mice (Jackson Laboratory, male, 4-6 weeks old) and tumor volume was calculated according to the previous study [43, 53].

### Immunohistochemistry assay

Immunohistochemistry (IHC) was used to detect TET2 gene expression in clinic HCC specimen and HCC cells. Primary antibody TET2 (Santa Cruz), 1:100 dilution.

### *In situ* hybridization assay

Procedure for ISH is as standard protocol [54]. DIG-labeled miR-22-3p probe (Exiqon). The expression of miR-22-3p in clinic HCC specimen was detected.

### Western blotting analysis

Procedure for Western blotting is as previous described [55]. TET2 (Santa Cruz), 1:500 dilution; β-actin (Protech, USA), 1:5000 dilution; GAPDH (Sigma, USA), 1:5000 dilution; Active β-catenin (Cell Signaling Technology, USA), 1:1000 dilution; E-cadherin/ N-cadherin/ Vimentin (Cell Signaling Technology, USA), 1:1000 dilution.

### Real-time q-PCR assay

The normalization control for miR-22-3p is U6 while the normalization control for TET2 is GAPDH. The information of the primers is shown in Supplementary Table 1.

### Statistical analysis

The data of this study was analyzed by SPSS16.0 software. Data are expressed as the mean ± SD of three independent experiments.

## Supplementary Material

Supplementary Figures

Supplementary Table 1
