# Correction for: Chronic alcohol exposure promotes HCC stemness and metastasis through *β*-catenin/miR-22-3p/TET2 axis

**DOI:** 10.18632/aging.206147

**Published:** 2024-10-31

**Authors:** Danlei Chen, Yan Yan, Xinyi Wang, Suzhi Li, Yan Liu, Dandan Yu, Yongjing He, Ruiqing Deng, Yakun Liu, Mei Xu, Jia Luo, Hongjun Gao, Siying Wang

**Affiliations:** 1 School of Basic Medical Sciences, Anhui Medical University, Hefei 230032, Anhui, China; 2 The First Affiliated Hospital of USTC, Division of Life Sciences and Medicine, University of Science and Technology of China, Hefei 230026, Anhui, China; 3 Department of Pulmonary Oncology, The Fifth Medical Center of Chinese PLA General Hospital, Fengtai, Beijing 100071, China; 4 Department of Pharmacology and Nutritional Sciences, University of Kentucky, College of Medicine, Lexington, KY 40536, USA; 5 Department of Pathology, University of Iowa Carver College of Medicine, Iowa City, IA 52242, USA

**Keywords:** HCC, metastasis, stemness, alcohol

**This article has been corrected:** The authors would like to correct the invasion assay image of the SMMC-7721 cells control group in **Figure 3L**. It was found that this image overlaps to a small degree the image of the SMMC-7721 miR-22-3p inhibitor group. This was the result of an unintentional error in the naming of the image file for the miR-22-3p inhibitor group. The authors provided the original data and corrected this error with an image from the original sets of experiments. This error does not affect the study’s conclusions, because the analysis was conducted using the correct datasets from repeated experiments.

The corrected version of **Figure 3** is provided below.

**Figure 3:**
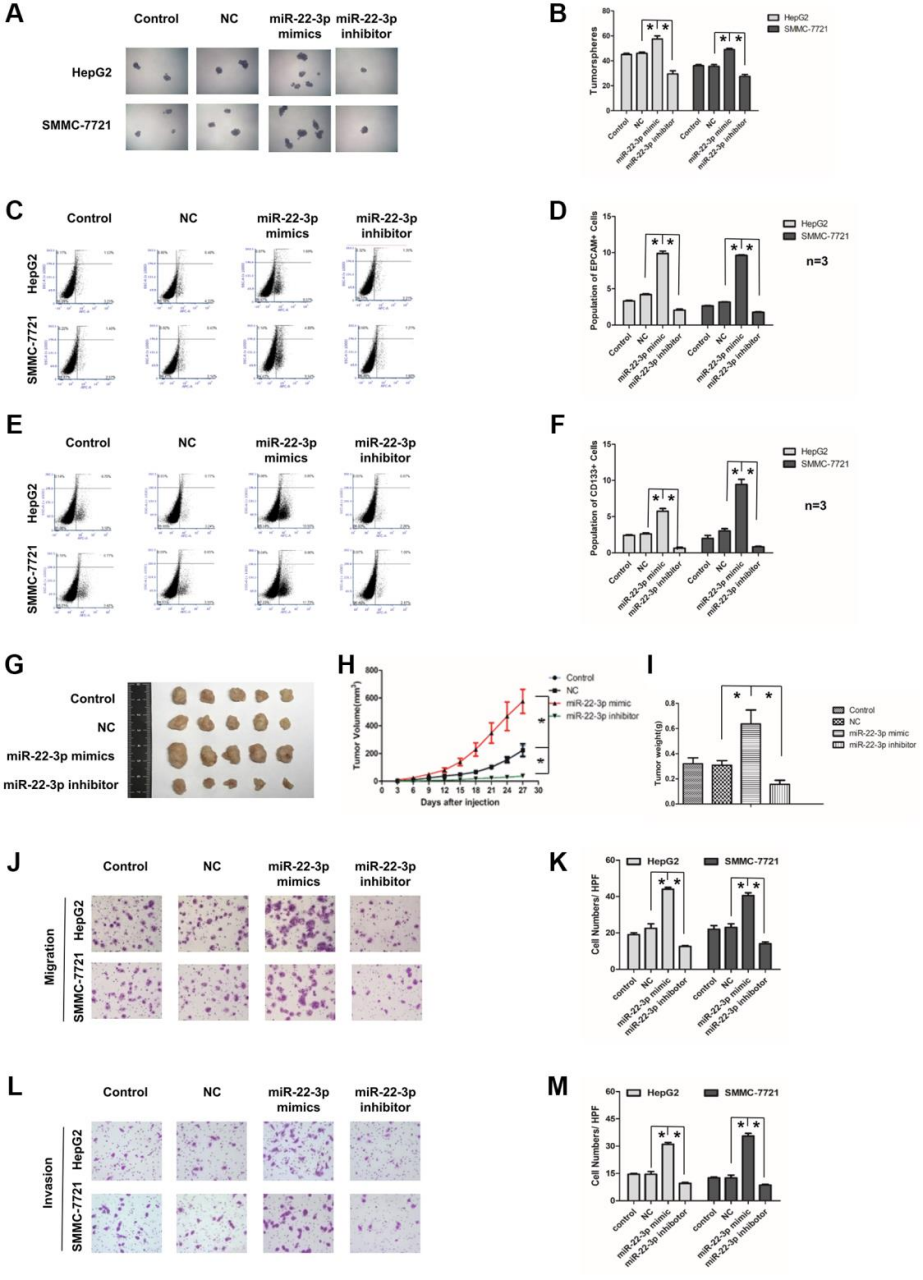
**Effects of miR-22-3p on stemness and metastasis of HCC cells.** (**A**) Tumorspheres formation ability of HCC cells in control, negative-control, miR-22-3p mimic and inhibitor groups. (**B**) The tumorspheres were quantified. ^*^P < 0.05. (**C**, **D**) Population of EPCAM- positive HCC cells in control, negative-control, miR-22-3p mimic and inhibitor groups. ^*^P < 0.05. (**E**, **F**) Population of CD133- positive HCC cells in control, negative-control, miR-22-3p mimic and inhibitor groups. ^*^P < 0.05. (**G**) The representative images of tumors taken from athymic mice inoculated with SMMC-7721 cells in control, negative-control, miR-22-3p mimic and inhibitor groups are shown. (**H**) The growth of tumor was calculated. Each group consisted of five mice. ^*^P < 0.05. (**I**) The tumor weight was quantified. Each group consisted of five mice. ^*^P < 0.05. (**J**) Representative image showing the migration of HCC cells in control, negative-control, miR-22-3p mimic and inhibitor groups. (**K**) The migrated cells were quantified. ^*^P < 0.05, *n* = 3. (**L**) Representative image showing the invasion of HCC cells in control, negative-control, miR-22-3p mimic and inhibitor groups. (**M**) The invaded cells were quantified. ^*^P < 0.05, *n* = 3.

