# Supplementary material for: Chronic alcohol exposure promotes HCC stemness and metastasis through β-catenin/miR-22-3p/TET2 axis
Source: Aging (Albany NY). 2021 May 21;13(10):14433–55. doi: 10.18632/aging.203059 (PMC8202861; doi:10.18632/aging.203059)
Supplement: Supplementary Figures [file aging-13-203059-s001.pdf]

SUPPLEMENTARY FIGURES

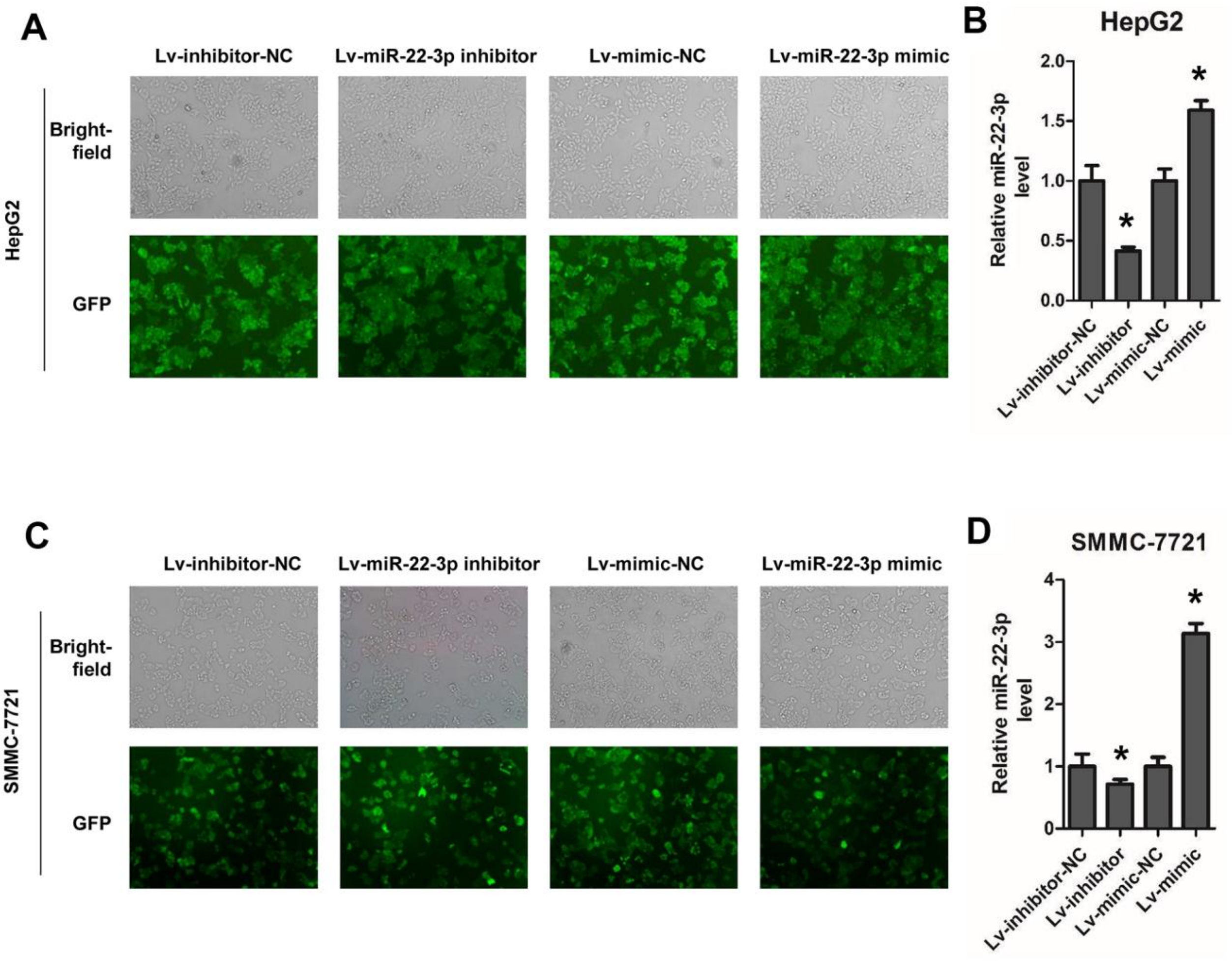

Supplementary Figure 1. Transfection efficiency of lentivirus miR-22-3p inhibitor and lentivirus miR-22-3p mimic. (**A**) GFP positive pictures showed the HepG2 cells successfully transfected with lentivirus miR-22-3p inhibitor and lentivirus miR-22-3p mimic. (**B**) Relative miR-22-3p level in HepG2 cells successfully infected with lentivirus expressing miR-22-3p inhibitor or miR-22-3p mimic. **P*<0.05. (**C**) GFP positive pictures showed the SMMC-7721 cells successfully infected with lentivirus expressing miR-22-3p inhibitor or miR-22-3p mimic. (**D**) Relative miR-22-3p level in SMMC-7721 cells successfully infected with lentivirus expressing miR-22-3p inhibitor or miR-22-3p mimic. **P*<0.05.

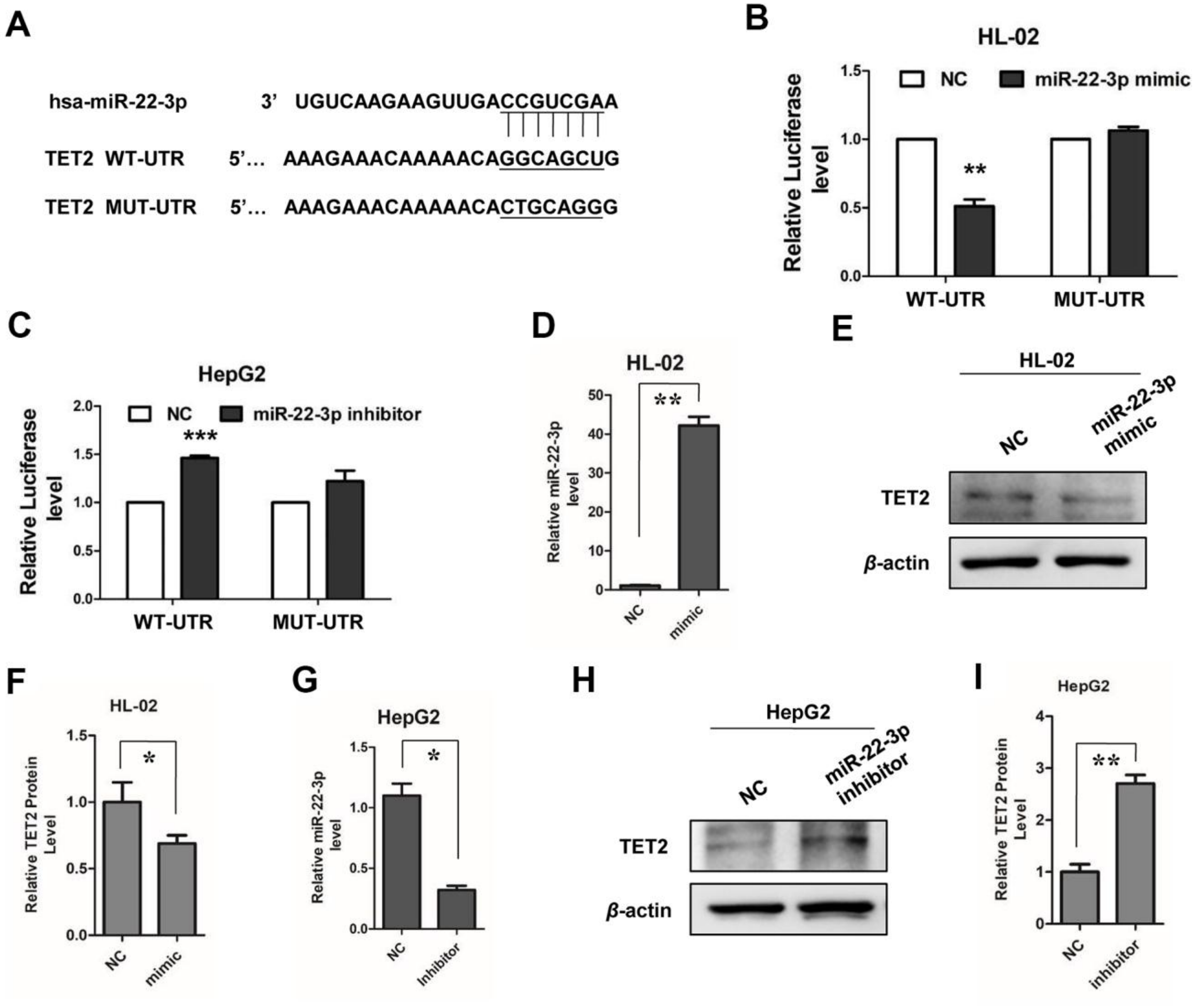

Supplementary Figure 2. TET2 is a direct downstream target of miR-22-3p. (**A**) Binding site of the TET2 3'-UTR and miR-22-3p. (**B**, **C**) Dual-luciferase activity of wildtype (WT) and mutant (MUT) TET2 3'-UTR reporter constructs in the presence of miR-22-3p. $^{**}P< 0.01$, $^{***}P< 0.001$. (**D**) miR-22-3p level in HL-02 cells transfected with miR-22-3p mimic. $^{**}P< 0.01$. (**E**, **F**) Protein level of TET2 in HL-02 cells transfected with miR-22-3p mimic. $^{*}P<0.05$. (**G**) miR-22-3p level in HepG2 cells transfected with miR-22-3p inhibitor. $^{*}P<0.05$. (**H**, **I**) Protein level of TET2 in HepG2 cells transfected with miR-22-3p inhibitor. $^{**}P< 0.01$.

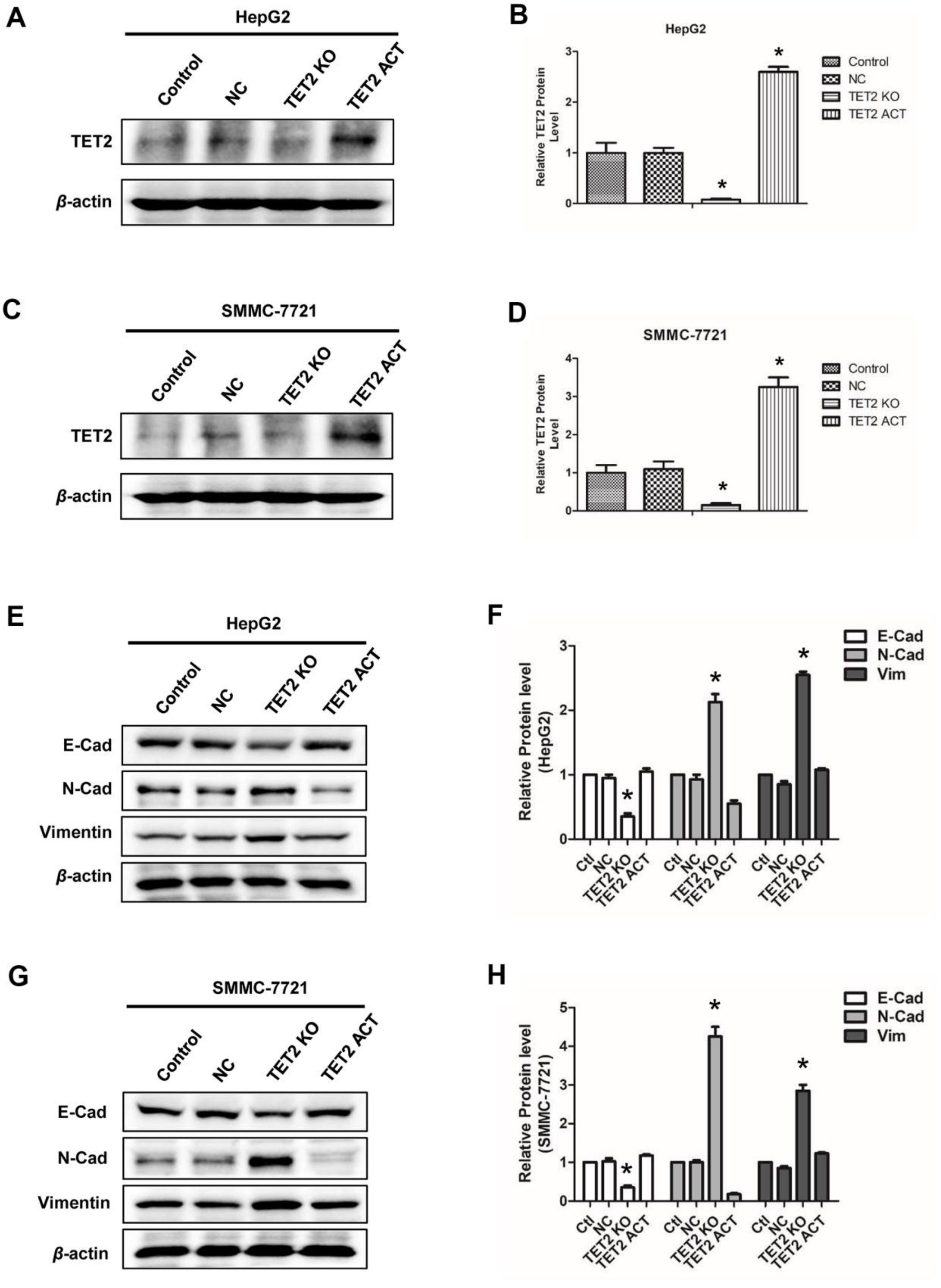

Supplementary Figure 3. Expression of EMT markers in HCC cells. (**A**–**D**) The protein level of TET2 in HCC cells transfected with TET2 CRISPR/Cas9Knockout (KO) and TET2 CRISPR/Cas9Activation (ACT) plasmid. **P*<0.05. (**E**–**H**) Expression of EMT related gene protein of HCC cells in control, negative-control, TET2 Knockout (KO) and TET2 Activation (ACT) groups. **P*< 0.05.

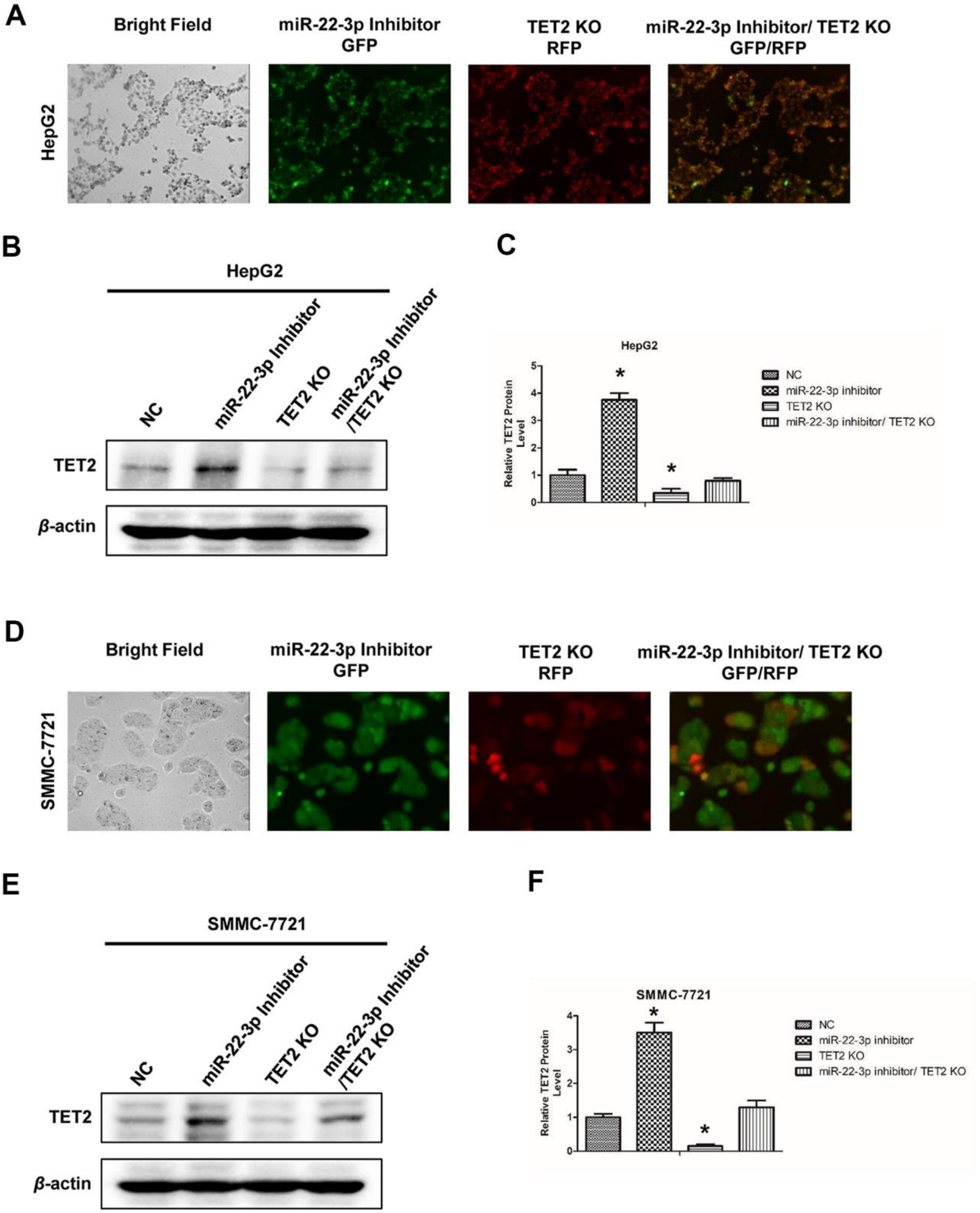

Supplementary Figure 4. Co-expression efficiency of lentivirus miR-22-3p inhibitor and TET2 CRISPR/Cas9 KO plasmid. (**A**) GFP and RFP positive pictures showed the HepG2 cells successfully infected with lentivirus expressing miR-22-3p inhibitor and transfected with TET2 CRISPR/Cas9 KO plasmid. (**B**, **C**) Relative TET2 level in HepG2 cells successfully infected with lentivirus expressing miR-22-3p inhibitor and transfected with TET2 CRISPR/Cas9 KO plasmid. **P*< 0.05. (**D**) GFP and RFP positive pictures showed the SMMC-7721 cells successfully infected with lentivirus expressing miR-22-3p inhibitor and transfected with TET2 CRISPR/Cas9 KO plasmid. (**E**, **F**) Relative TET2 level in SMMC-7721 cells successfully infected with lentivirus expressing miR-22-3p inhibitor and transfected with TET2 CRISPR/Cas9 KO plasmid. **P*< 0.05.

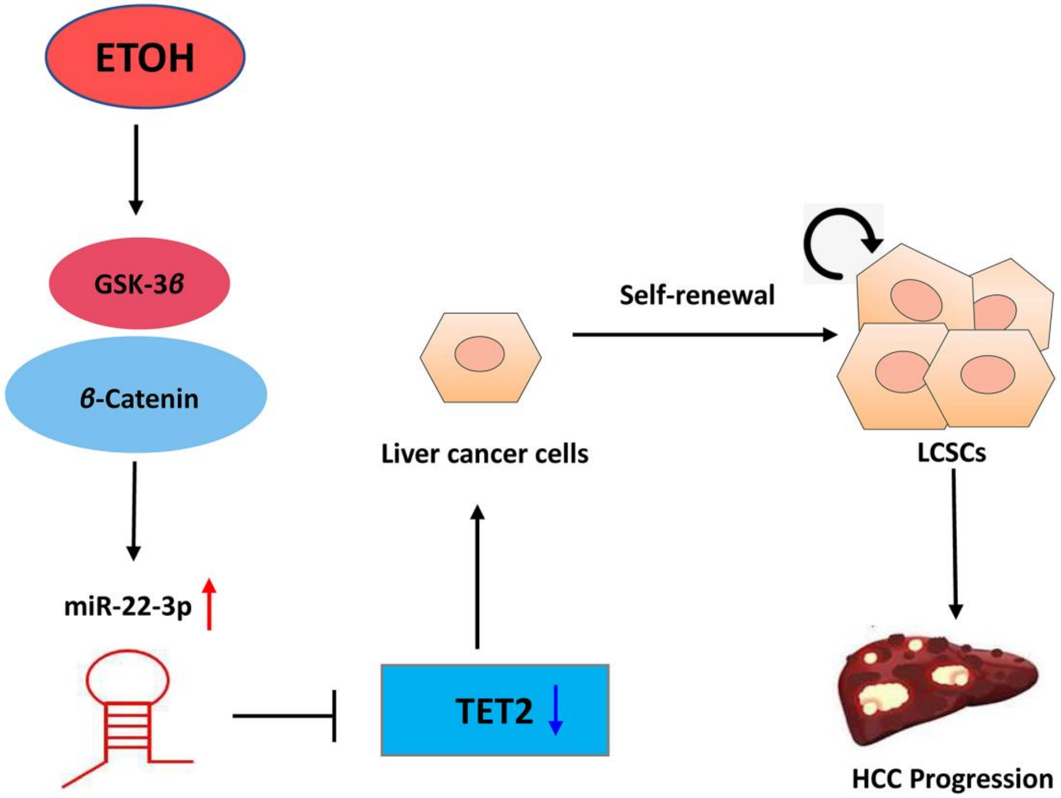

Supplementary Figure 5. Working model of β-catenin/miR-22-3p/TET2 axis. Chronic ethanol exposure promotes HCC progression through β-catenin/miR-22-3p/TET2 axis.
