# Supplementary material for: Chronic alcohol exposure promotes HCC stemness and metastasis through β-catenin/miR-22-3p/TET2 axis
Source: Aging (Albany NY). 2021 May 21;13(10):14433–55. doi: 10.18632/aging.203059 (PMC8202861; doi:10.18632/aging.203059)
Supplement: Supplementary Table 1 [file aging-13-203059-s002.pdf]

SUPPLEMENTARY TABLE

Supplementary Table 1. Information of primers used in this study.

miR-22-3p F Primer: TATA GTAG AAAG CTGC CAGT TGAA G
miR-22-3p R Primer: TATG GTTG TTCT GCTC TCTG TGTC
U6 F Primer: ATTG GAAC GATA CAGA GAAG AT
U6 R Primer: GGAA CGCT TCAC GAAT TTG
TET2 F Primer: ATAC CCTG TATG AAGG GAAG CC
TET2 R Primer: CTTA CCCC GAAG TTAC GTCT TTC
GAPDH F Primer: ATGG GGAA GGTG AAGG TCG
GAPDH R Primer: GGGG TCAT TGAT GGCA ACAAT A
